# From Social Network (Centralized vs. Decentralized) to Collective Decision-Making (Unshared vs. Shared Consensus)

**DOI:** 10.1371/journal.pone.0032566

**Published:** 2012-02-29

**Authors:** Cédric Sueur, Jean-Louis Deneubourg, Odile Petit

**Affiliations:** 1 Unit of Social Ecology, Université Libre de Bruxelles, Campus Plaine, Bd du Triomphe, Brussels, Belgium; 2 Centre National de la Recherche Scientifique, Département Ecologie, Physiologie et Ethologie, Strasbourg, France; 3 Université de Strasbourg, Institut Pluridisciplinaire Hubert Curien, Strasbourg, France; King Abdullah University of Science and Technology, Saudi Arabia

## Abstract

Relationships we have with our friends, family, or colleagues influence our personal decisions, as well as decisions we make together with others. As in human beings, despotism and egalitarian societies seem to also exist in animals. While studies have shown that social networks constrain many phenomena from amoebae to primates, we still do not know how consensus emerges from the properties of social networks in many biological systems. We created artificial social networks that represent the continuum from centralized to decentralized organization and used an agent-based model to make predictions about the patterns of consensus and collective movements we observed according to the social network. These theoretical results showed that different social networks and especially contrasted ones – star network vs. equal network - led to totally different patterns. Our model showed that, by moving from a centralized network to a decentralized one, the central individual seemed to lose its leadership in the collective movement's decisions. We, therefore, showed a link between the type of social network and the resulting consensus. By comparing our theoretical data with data on five groups of primates, we confirmed that this relationship between social network and consensus also appears to exist in animal societies.

## Introduction

Every day humans make decisions. Any decision made by an individual is influenced by the relationships he or she has with people in different circumstances. For example, the head of a firm, or the parents in a family, may exert greater influence in the decision-making process than other contributors do. For a presidential election, people generally decide individually which candidate they will vote for, even if friends and family may influence their decision to a certain extent. Broadly speaking, two systems of decision-making are often described: on the one hand, one individual, or one sub-group, decides for the rest of the group (*unshared consensus*, [1]); on the other hand, each individual can make independent decisions and take an equal part in the vote (*shared consensus*, [1]). This study aimed to understand how the social network – the structure of social relationships between the members of a group - may affect the influence of these individuals on collective decision-making and thus lead to unshared or shared consensus [1]–[4].

*Consensus decision-making* in animal groups has already been described by several authors (for a review, see [1]). Many animal species live in groups and have to reach consensus in order to maintain cohesion [5]. One of the most tractable ways of understanding how group members attain consensus is to study collective movements [6]–[9]. In this context, consensus decision-making for group movements has been described as a continuum – from an *unshared consensus* to an *equally shared consensus* [1], [10].

The influence of ecological constraints has often been used to explain the type of consensus observed [11], [12]. Studies have reported that specific individuals lead groups with the aim of gaining better personal access to food (*Papio ursinus* [9]; *Equus burchellii* [13]; *Pan troglodytes* [14]). In other studies, individuals who know where to find the best food resources can become the leaders [15]. These two general cases – leading according to needs or according to knowledge - can be qualified as unshared or partially shared consensus. On the other hand, shared consensus allows information to be pooled and may lead to more appropriate decisions for all group members [7], [16].

Nevertheless, these previous studies did not explore the influence that social network could have on the decision-making process. This type of direct link between the properties of social networks and the kind of consensus has been suggested [1], [10], [17], but has never been empirically tested. Moreover, as stated in [18], “models of collective motion typically do not consider social network structure”, despite the fact that an increasing number of studies illustrate how group social networks are both complex and crucial for understanding the synchronisation of group activity. Some authors have modelled the effect of social network on collective motion in human crowds [19] and in fish shoals [20], but their results were not compared to empirical data and the social networks used in their model were not representative of networks observed in animal groups. Indeed, they used simple networks, whereas Erdos-Renyi random networks or scale-free networks were found in several animal species (see [17], [21]–[24]). These social networks described in animals may constrain many social phenomena such as information or disease transmission, cooperation and group fission in species ranging from amoebae to primates [25]–[30]. The strength of the social relationships of group members is not only based on ecological constraints but also on species-specific and group-specific internal factors [31]. In the genus *Macaca* [32] or *Cebus* [33], different social styles [34] have been described, ranging from despotic to egalitarian societies [35]. Vehrencamp [36] first described these societies as follows: “Variation in the balance point between the forces of cooperation and competition is common from society to society. In egalitarian societies, benefits are divided roughly equally or in proportion to the risk or effort taken. In despotic societies, on the other hand, benefits accrue disproportionately to a few individuals in the group at the expense of others. Societies can thus be ranked along a continuum in terms of the degree to which fitnesses of individuals within social groups are biased”. This variation among social networks can be observed through behavioural patterns that co-vary [32]. In rhesus and Japanese macaques (*Macaca mulatta* and *M. fuscata*), for example, most conflicts are unidirectional, high-intensity aggression is common, and few conflicts are reconciled. The dominant male appears to be very central, managing conflicts and receiving the most grooming or other affiliative interactions [21]. In Sulawesi macaques, most conflicts are bidirectional, aggression is generally of low intensity, conciliatory tendencies are frequent and grooming is distributed between all individuals rather than centralized on the dominant male [31]. There is a recursive feedback loop between the social network and individual behaviour. Sueur and Petit [10] suggested a similar link between social networks, - especially centrality - and consensus in their studies on collective movements in macaques. An equally shared consensus was found in the egalitarian Tonkean macaques (*M. tonkeana*), whereas the more despotic rhesus macaques used a partially shared consensus when deciding to move. In the same way, it has previously been reported that species with strict hierarchies appear to have unshared, or partially shared, consensus (*Canis lupus* [37]; *Helogale parvula* [38]; *Equus caballus* [39]; *Gorilla gorilla berengei* [40]).

Within many such societal organizations – despotic or egalitarian - it is still not known how consensuses (reaching a common decision in spite of conflicts of interest) emerge from then influence properties of the social network. These networks, despotic and egalitarian can be directly compared to centralized and decentralized networks respectively [41]. Dominant or central individuals are classically described as leaders but many factors may constrain this leadership and we do not know if social relationships really influence consensus, nor which of these relationships (aggressive *vs.* affiliative for instance) influences the consensus and to what extent it does so. Here, we based our study on the assumption that the extent of affiliative relationships may lead to a specific type of consensus, as the distribution of these relationships seems to drive many other phenomena. However, to test this assumption, we first need to combine an experimental approach on several groups with modelling, and then have to combine social network analysis with models for collective motion. We first created artificial social networks – representing the continuum from centralized to decentralized organization – and then developed a stochastic model to make predictions about the patterns of collective movements that would emerge from them. Who leads? And who is more successfully followed, both in terms of the number of followers and of the time needed for a follower to join the movement? We predict that the more centralized the network is, the more differences will appear between individuals, with the emergence of a leader during collective movements. And if it has a central position in the network, this leader will increase both the number of joiners and the joining speed of individuals. We then compared the relations between social network and patterns of collective movements to linear and non-linear functions in order to establish how leadership emerges from the social network. Indeed, many studies have already shown that the relation between the information transfer and the probability of performing a behaviour does not increase linearly but in a non-linear way due to an amplification process [42]–[44]. In order to validate or nullify our assumptions based on simulations, the theoretical data was therefore compared to observed data collected from the observation of collective movements in five different groups of primates living in similar semi free-ranging conditions.

## Methods

### Ethics Statement

This study involved the observation of animals without animal handling or invasive experiments carried out on studied subjects. We declare that our study was carried out in full accordance with the ethical guidelines of our institution with the approval of the latter (certificate number: 67-339, French Republic, Bas-Rhin County Hall, French veterinary services). Our experiments comply with European animal welfare legislation. The work being carried out during this study is in accordance with the weatherall report and all efforts were made to ensure the welfare of the animals and minimize suffering. Concerning the amelioration of animal welfare, the study groups were bred under semi free-ranging conditions at the Strasbourg University Centre of Primatology. They had complete access to about 0.35 ha (maximal length = 80 m; maximal width = 60 m) of wooded parkland as well as indoor housing within the enclosure. The indoor housing (20 m^2^) is made of cement and tiling. The enclosure area was made up of various slopes and uneven ground. The distribution of vegetation was also heterogeneous, with three layers (grass, trees and bushes) that were unevenly distributed throughout the enclosure. For each group, fresh fruit and vegetables were provided once a week, one hour after the end of the observation session. Thus, the behaviour of the animals was unlikely to be affected by this event. Animals were used to human presence in their enclosure.

### Modelling

The model is based on rules of mimetism/cohesion (Markov chain process) described in several studies on collective phenomena [42]–[44]. In this model, the probability that an individual will join the collective movement depends on the number but also the strength of relationships it has with the individuals already participating in the movement. The number of individuals, individual identities and the network of affiliative relationships of each artificial social network are included in the model. At the start of a simulation, all agents (*N*) were in an area called the resting area and had to move to another area, the foraging area. We implemented the intrinsic probability *λ_i_* of each agent. This intrinsic probability is independent of the influence of conspecifics, and is, for example, a nutrient need. The departure probability of the initiator (first individual to depart) was the same whatever the social network and identity of this individual and was constant per time unit. The departure probability *ψ_01_* of the initiator was:
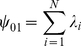
(1)


The probability *ψ_i_* of an individual *i* becoming a joiner *j* was:
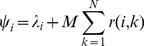
(2)where λ*_i_* is the intrinsic probability of each agent (*λ_i_* = 0.00007), *M* is the mimetic coefficient (*M* = 0.002), and r*(i,k)* is the affiliative relationships between the resting individual *i* and the already moving individual *k*. If *k* was not moving, *r(i,k)* = 0. Values of *r(i,k)* are given in the section “artificial social networks”. According to the values of *λ_i_* and *M*, *ψ_i_*<1.

We implemented the model and each artificial social network in Netlogo 3.1.4 [45], [46]. At each time step (corresponding to 1 second in the real system), a number between 0 and 1 was randomly attributed to each resting agent (i.e. in the resting area); when this number was lower than the theoretical departure probability of each agent, the individual left the resting area for the foraging area; if this number was higher than the theoretical departure probability, the agent did not move. To be consistent with observed data [3], [8], [47]–[51], we stopped a simulation when no agent joined within 300 s of the departure of the initiator or the last joiner. We set the number of simulations at 10,000 for each social network.

### Artificial social networks

**Figure 1:**
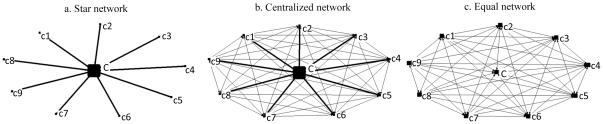
Graph representation of social networks. (a) Star network (extremely centralized), (b) centralized networks (highly, intermediately, and low), and (c) equal network (extremely decentralized). Squares represent individuals. *C* is the central individual, c_1–9_ are non central individuals. Lines are relationships between individuals: the thicker the line, the stronger the relationship. The size of square represents the eigenvector centrality: the bigger the square, the higher the centrality.

A group can be defined as a network in which each dyad of individuals is characterized by one or several types of social bonds such as hierarchical, kin or affiliative ones [52], [53]. In this study, in order to gain insight into the generic properties of how social relationships result in consensus decision-making, we designed a deliberately simplified social structure, and thus only investigated affiliative relationships related to movement decisions. We created artificial networks consisting of 10 individuals (*N* = 10), this group size being close to that of the five observed primate groups. Links, i.e. affiliative relationships, can be calculated by scoring proximities, contacts, or grooming duration between individuals [25], [54]. Each individual *i* has a fixed quantity of social interactions (*Σr(i,k)* = 1) that it can divide between its conspecifics. Indeed, several studies have showed that social interactions are time-constrained and seemed to be maintained at a certain value, whatever the group size or the environmental pressures [26], [55]. Each individual *k* will receive an amount of social interactions *r(i,k)* from the individual *i*. As the main topic of our study is based on the impact of the central individual (i.e. the strength of centrality) on collective decision-making, we studied how the relationships between all non central individuals *c* and the central individual *C* influenced the patterns of decision-making during collective movements, i.e., who is the most successful individual in terms of number of followers and how rapidly followers join the movement. For each social network included in this study, the central individual will be considered the individual having the most numerous and strongest relationships (based on the eigenvector coefficient). This study aimed to observe different patterns of collective decision-making according to the distribution of relationships within the group. Each individual has one social relationship *r(i,k)* with each of the *N−1* other group members. We defined (1) *r(i,k)* = *r(c,C)*, the relationship that a non central individual *c* had with the central individual *C*; (2) *r(i,k)* = *r(c,c)*, the relationship that a non central individual *c* had with any other non central individual *c* (for each network, all *r(c,c)* are equal); and (3) *r(i,k)* = *r(C,c)*, the relationship that the central individual *C* had with any non central individual *c*. We created different social networks, from extremely centralized to extremely decentralized, by varying the relationship that an individual *i* had with a congener *k* and especially the relationships that non central individuals *c* had with the central individual *C*. For instance, we attributed to *r(c,C)* a value equal to 1 for the extremely centralized system (called the *star network*) and equal to *1/(N−1)* for the extremely decentralized system (called the *equal network*) ([41], [54]). Whatever the network, however, *r(C,c)*, the value of interaction attributed by the central individual *C* to each non central individual *c*, equalled *1/(N−1)*. We eventually created six different social networks where *r(c,C)* and then *r(c,c)* differed (see Table 1 for detailed values of each network and Fig. 1A–C for network representations). As all *r(i,k) = 1* and then *Σr(i,k)* = 9 for each network, we can compare all networks together in analyses. We also built a random network (Erdos-Renyi graph), which was obtained using Ucinet 6.0 [56] and a chain network. Random network is found in several primate species [23], [57] but the chain network is a hierarchical network only found in human beings and more specifically in military departments or in firms [41], [53]. Graphs of these networks are given in supplementary material (Fig. S1), and their indices can be found in Table S1.

**Table 1:** Relationships, eigenvector centrality for the central individual *C* and the non central individuals *c*, and centrality index for each social network.

Network	r(c, C)	r(c, c)	r(C, c)	Eigenvector of C	Eigenvector of c	Centrality index
Star	1	0	1/(n–1)	0.95	0.1	0.85
Highly centralized	0.75	0.25/(n–2)	1/(n–1)	0.91	0.14	0.77
Intermediatelycentralized	0.50	0.50/(n–2)	1/(n–1)	0.83	0.18	0.65
Low centralized	0.25	0.75/(n–2)	1/(n–1)	0.6	0.27	0.33
Very low centralized	0.125	0.875/(n-2)	1/(n-1)	0.36	0.31	0.05
Equal	1/(n–1)	1/(n–1)	1/(n–1)	0.32	0.32	0

According to equation 2 of our model, the more a resting agent had strongly affiliated moving agents, the greater its probability of joining was. Thus, the more a moving agent had strongly affiliated resting agents, the greater its probability of being joined was. This means that in the *equal network*, the probability of joining the movement would only depend on the number of agents already moving, whatever their identities. Conversely, in the *star network*, as *r(c,C)>r(C,c)*, the central individual C would have more probability of being joined than any non central individual c.

### Empirical data

In order to validate our theoretical results, we compared them to observed data. We used affiliative relationships (based on body contacts or proximities between individuals) in relation to movements and collective decision-making patterns during the collective movements of five different groups of primates living in similar semi free-ranging conditions. Data were obtained from two groups of Tonkean macaques [3], [8], [17], [50], [51], one group of rhesus macaques [3], [8], [17], [50], [51], one group of brown lemurs (*Lemur macaco*, [47], [58]) and one group of white-faced capuchin monkeys (*Cebus capucinus*, [33], [48], [49]). All authors used similar data scoring and similar definitions for collective movements and affiliative relationships, minimizing the risk of methodological bias in the comparisons.

As the distribution of the first departure latencies (time between the end of the previous collective movement and the departure of the initiator of the new collective movement [3], [47], [49]) corresponded to an exponential distribution in the observed groups [3], [47], [49], we used the mean log gradient of this exponential distribution, that is, the inverse of the mean departure latency of the initiator [3], [43], [43], [47,49], to calculate the departure probability *ψ_01_* of the initiator. *ψ_01_* = 0.0007 for any initiator of any social network.

Then in our model, given that *ψ_01_* = 0.0007 per s and *n* = *N* = 10, the probability per individual of departing first is λ_i_ = 0.00007 per s. In our case, the probabilities were identical with λ_1_ = … = λ_n_ = λ, meaning that all individuals had the same intrinsic probability.

Since the distribution of the inverse of joiner departure latencies (time between the departure of the previous joiner *j−1* and the departure of the joiner *j*) of each observed group fitted a parabolic curve [3], [43], [43], [47,49], we could implement a mimetic process (i.e. *M*) in our model, representing an amplification of the probability of joining a movement with the number of individuals already participating in this movement. The calculation of the mean mimetic coefficient *M* was based on that found in five primate groups and this coefficient equals 0.002.

### Data scoring and calculation

#### Indices of social networks

Affiliative relationships among observed groups were determined using proximities/contacts between individuals [3], [17], [33], [47]–[50], [58]. We determined the *eigenvector centrality coefficient* for each individual in each artificial social network and each observed group (i.e. real group). This index takes into account the number and the strength of relationships between an individual and its conspecifics, as well as the relationships its associated individuals have with other group members. On the contrary, indices such as the clustering coefficient or the betweenness coefficient do not take into account the strength of relationships but only their number [52]. For the *equal network*, all eigenvector centrality coefficients were equal whatever the individual identities (see Table 1 for details). In the *star network* and the three different *centralized networks*, the coefficients of non central individuals *c* are equal to one another but smaller than that of the central individual *C*. We then calculated the difference between the eigenvector centrality coefficient of the central individual *C* and the mean eigenvector centrality coefficient of all non central individuals *c*. This index, which we called the *centrality index*, allowed us to quantify the degree of centrality or *centralization* [59] of a social network – the group – and to compare the artificial social networks with the observed groups [32]. Indeed, this index allows us to quantify the difference of centralities between the central individual and the other individuals in the group. This index ranged from 0 – in a decentralized network where all individuals have the same relationships – to 1 in a centralized network where group members only have social relationships with the central individuals. In this study, the *centrality index* varied from 0 for the *equal network* to 0.85 for the *star network* (see Table 1 for details).

#### Data about collective movements

The number of joiners was scored for each collective movement. We also scored the identity and the departure latency of the initiator (ΔT*_01_*) and of every joiner (ΔT*_j−1,j_*). We calculated the rank of each agent during the joining process, regardless of its identity. The rank of the initiator was rank 1, the rank of the first joiner was rank 2, and the rank of the *j* ^th^ joiner was rank *j+1*. Departure latency of the initiator ΔT*_01_* was calculated by scoring the time elapsed between the start of the simulation and the departure of this individual. We then scored the departure latency of each joiner, that is, the departure latency of the joiner *j*, ΔT*_j−1,j_*, corresponding to the time elapsed between the departure of the joiner *j−1* (i.e. the previous departing individual, including the initiator) and the departure of the joiner *j*. Finally, we scored the duration of joining ΔT*_1,10_* as the time elapsed between the departure of the initiator and the departure of the last joiner only when all individuals (*N* = 10) joined the movement.

For (1) the number of joiners *n*, (2) the departure latency of the first joiner ΔT_1,2_, and (3) the duration of joining ΔT*_1,10_*, we calculated the average 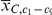 for all individuals, the average 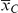 for the central individual *C*, and the average 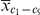
for the non central individuals *c*, when these individuals initiated movements. These were the main three variables for our analysis. For each of these three variables, we then calculated the absolute difference between the central individual *C* and the non central individuals *c*, 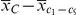. We also assessed whether or not the number of joiners was different when the central individual *C* was the first joiner.

### Statistical analyses

Data on random network and chain network were only included in *Global analyses* in order to avoid any interference with our main aim, which was to understand the relationship between social networks and collective decision-making by studying the role of the central individual.

*Global analyses:* We first analysed the possible link between the *centrality index* and the average 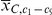
of the three main variables: the mean number of joiners 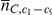, the mean departure latency of the first joiner 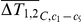, and the mean duration of joining 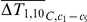, using curve estimation tests. The curve estimation test determined the best relation [60], [61] between two variables (linear, exponential and logarithmic) to understand if the leadership emerges in a linear or non-linear way from the properties of the social networks. We assessed whether differences exist between each artificial social network using a Kruskal-Wallis test followed by a Dunn's multiple comparisons test. We also compared the departure latency distribution of each social network using Spearman rank correlation tests. The initiator's latencies were not included in this test because, as explained above, the departure probability of this individual was identical whatever the social network.

*Differences between the central and non central individuals*: We first analysed how the difference 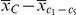 varied according to the social network for the three variables (i.e. 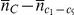; 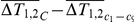; 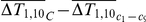) using curve estimation tests. Next we assessed whether the three variables (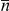,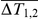,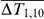) were different between the central individual *C* and the non central individuals *c* using Mann-Whitney tests (number of data equals 10,000 for each condition). We then determined whether or not a difference in the number of joiners existed when the central individual *C* was the first joiner for each social network (using a Mann-Whitney test). Finally, departure latency distribution of the central individual *C* was compared with those of the non central individuals *c* in each social network using a Spearman rank correlation test.

#### Comparisons of theoretical and observed data

The values obtained for the three main variables in the simulations were compared with those observed in the five primate groups. Using a linear curve estimation test, we assessed for similar *centrality indices* whether we obtained a correlation between the simulated data and the observed data for the differences 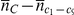, 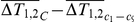, and 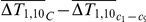.

We carried out the statistical tests in SPSS 10.0; α = 0.05. Means are ± SE (standard error).

## Results

### 1) Global analyses

These global analyses will highlight differences in decision-making between the different social networks tested.

#### Mean number of joiners

**Figure 2:**
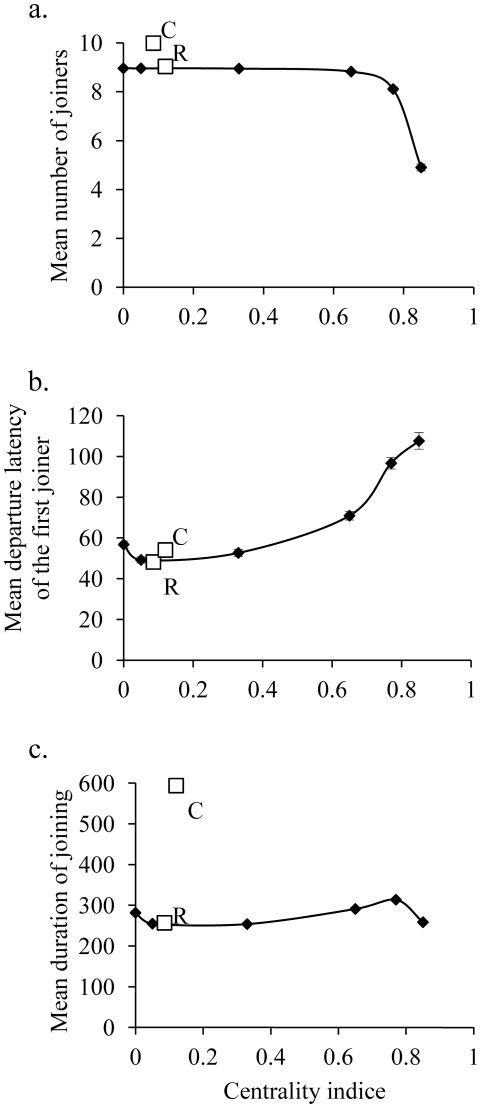
Relation between the centrality index and variables of collective decisions (movements). (a) Mean number of joiners, (b) mean departure latency of the first joiner, and (c) mean duration of joining whatever the initiator's identity. The relation between the centrality index and the mean duration of joiners follows an exponential law. The relation between the centrality index and the mean departure latency of the first joiner does not follow any tested law. The relation between the centrality index and the mean duration of joining follows an exponential law. Parameters of functions are given in the global analyses section of Results. For each network, error bars are indicated and represent the standard error. R indicates the result for random network and C indicates the result for chain networks.

We investigated the relation between the mean number of joiners 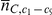
and social network type (from centralized to decentralized). The test revealed that the curve best followed an exponential inverse law (R^2^ = 0.81, F_1,4_ = 17.02, p = 0.015; y = 0.0263e^4.6039(x-9)^) and Figure 2A suggested a sudden decrease for *high decentralized* indices. The mean numbers of joiners of the *star* and *highly decentralized* networks were lower than those of the other networks (K-W: H = 1789, df = 7, p = 0.0001; Dunn's test: p<0.0001, see Table S2 in the supplementary material for details). However, the number of joiners in the *chain network* was higher than that observed in other networks.

#### Mean departure latency of the first joiner

The mean departure latency of the first joiner 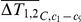 increased with the *centrality index* in an exponential way (R^2^ = 0.82, F_1,4_ = 18.09, p = 0.013, y = 48.37e^0.81x^; Fig. 2B). Indeed, this departure latency was higher for the *star* and the *highly centralized* networks than for all other networks (K-W: H = 239.6, df = 7, p = 0.0001; Dunn's multiple comparisons test: p<0.0001).

#### Mean duration of joining

The mean duration of joining 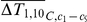 did not varied according to the type of social network (R^2^<0.14, F_1,4_ = 0.64, p>0.466, Fig. 2C). However, the mean duration of joining was higher for the *chain* and *highly centralized* networks than for the other networks (K-W: H = 234.2, df = 7, p = 0.0001; Dunn's multiple comparisons test: p<0.01).

#### Latency distributions

We assessed whether the joining of each individual to the movement differs according to the social network. We studied latency distribution according to rank of joining and then compared the distributions of each network. The latency distribution in the *star network* was not correlated with the distributions found in the other networks (*star network* vs. other networks: rs <0.28, N = 9, p>0.058) whilst all other networks were correlated with each other (other networks between them: rs >0.83, N = 9, p<0.005; see Fig. S2 in the supplementary material information).

### 2) Differences between the central individual and the non central ones

These analyses assessed how the probabilities of joining the movement, and thus the speed of decision-making and the number of joiners, are affected by the identity of the initiator.

#### Mean number of joiners

**Figure 3:**
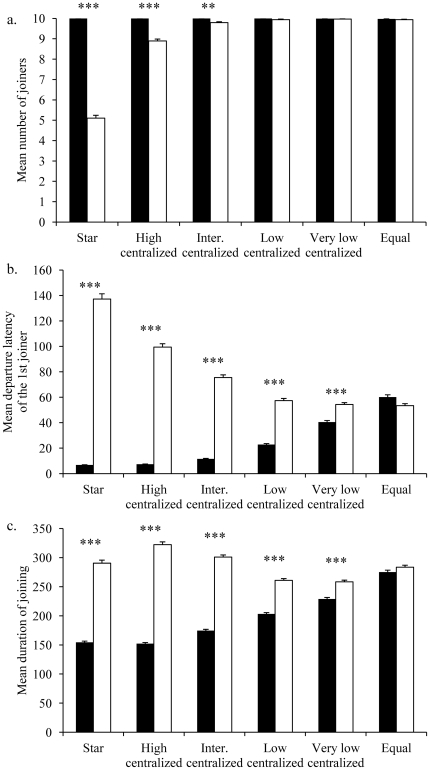
Comparison between the central individual and the non central individuals. (a) Mean number of joiners, (b) mean departure latency of the first joiner, and (c) mean joining duration for each social network. Central individual is represented by the black bars, non central individuals are represented by the white bars.

We first checked how the difference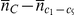 varied according to social network. The curve considered followed an exponential law (R^2^ = 0.77, F_1,4_ = 10.41, p = 0.03, y = 0.003e^7.75x^). The higher the *centrality index* of the initiator was, the higher the difference in the number of joiners between the central individual *C* and the non central individuals *c* was. This result was confirmed by Mann-Whitney tests which showed that the difference in the mean number of joiners between the central individual *C* and the non central individuals *c* was only significant for the *star* (Z = −27.17, p<0.0001, 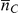 = 9.99, 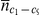 = 5.1), *high centralized* (Z = −11.22, p<0.0001, 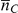 = 9.99, 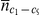 = 8.89), and *intermediately centralized* (Z = −3.7, p = 0.0002, 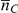 = 9.99, 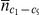 = 9.79) networks. The number of joiners decreased with the *centrality index* when non central individuals were initiators (Fig. 3A).

In the same way, when the central individual *C* was the first joiner, this increased the number of individuals joining a movement for the *star* (Z = −7.3, p<0.0001, 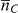 = 9.99, 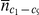 = 8.15) and *highly centralized* (Z = −2.2, p = 0.028, 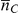 = 10, 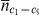 = 9.8) networks. There was no such increase for the remaining networks because movements initiated by non central individuals *c* already had approximately 10 joiners, even if the first joiner was not the central individual *C*.

#### Mean departure latency of the first joiner

The curve estimation test showed that the difference 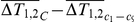 varied according to a linear law with the *centrality index* (R^2^ = 0.93, F_1,4_ = 51.11, p = 0.002, y = 19.293x+0.6889). This difference was significant for the *star* (Z = −32.9, p<0.0001, 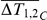 = 6.75, 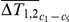 = 137.22), *highly centralized* (Z = −29.3, p<0.0001, 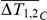 = 7.35, 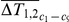 = 99.4), *intermediately centralized* (Z = −29.7, p<0.0001, 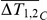 = 11.6, 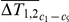 = 75.5), *low centralized* (Z = −17, p<0.0001, 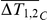 = 22.9, 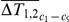
57.3) and *very low centralized* (Z = −7.5, p<0.0001, 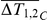 = 40.5, 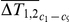 = 54.24) networks. The departure latency of the first joiner decreased with the *centrality index* when the central individual *C* initiated the movement, whilst it increased when a non central individual *c* initiated it (Fig. 3B).

#### Mean duration of joining

The difference 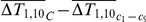 increased with the *centrality index* according to a linear curve (R^2^ = 0.92, F_1,4_ = 46.14, p = 0.002). This difference was statistically significant for non-equal networks (Z<−14.7, p<0.0001 for all networks; *star*: 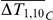 = 154.4, 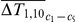 = 290.5; *highly centralized*: 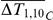 = 152.2, 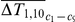 = 322.4; *intermediately centralized*: 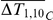 = 174.7, 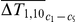
300.9, *low centralized:* 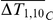 = 203.3, 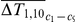
261.6, *very low centralized*: 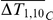 = 228.5, 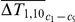 = 261.3). The mean duration of joining greatly decreased with the *centrality index* when the central individual *C* initiated movements (Fig. 3C).

#### Latency distributions

For each social network, we assessed whether latency distribution differed according to whether movements were initiated by the central individual *C* or the non central individuals *c*. On the one hand, the two latency distributions were correlated for the *low centralized* (rs = 0.73, N = 9, p = 0.02), *very low centralized* (rs = 0.95, N = 9, p<0.0001) and *equal* (rs = 0.99, N = 9, p<0.0001) networks (Fig. S3); the response and the probability of joiners were similar for these three networks, whatever the initiator's identity. On the other hand, they were not correlated for the *star* (rs = 0.3, N = 9, p = 0.432), *highly centralized* (rs = −0.31, N = 9, p = 0.406), or *intermediately centralized* (rs = −0.13, N = 9, p = 0.732) networks (Fig. S3).

### 3) Comparisons between the artificial and observed social networks

These comparisons with observed decision-making in natural primate groups were conducted in order to validate our theoretical results.

#### Mean number of joiners

We determined whether for similar *centrality indices* we obtained a correlation between the simulated data and the observed ones for the difference 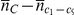. The two variables were correlated (R^2^ = 0.83, F_1,3_ = 57.6, p = 0.03). We obtained the same relation between the number of joiners and the *centrality index* for theoretical and observed data. The difference between the central individual *C* and the non central individuals *c* was, however, about 100-fold higher in the observed data compared to theoretical data.

#### Mean departure latency of the first joiner

Contrary to results above, there was no correlation between observed and theoretical data for the difference 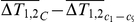 (R^2^ = 0.04, F_1,3_ = 0.124, p = 0.747)

#### Mean duration of joining

The results showed a correlation for the difference 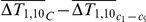 (R^2^ = 0.95, F_1,3_ = 15.3, p = 0.003). We obtained the same relation between the mean duration of joining and the *centrality index* in theoretical and observed data.

## Discussion

For a long time, scientists have tried to understand the origins of leadership in non-human primates and humans [1], [4], [7], [62]. In this study, we assessed how social networks from centralized to decentralized can affect collective decisions and specifically the emergence of this leadership. By analysing patterns of collective decision-making such as joining speed, we aimed to determine whether animals gain an advantage - in terms of saving time or information transfer - by adopting one social network or another. Our results showed that different social networks, and especially opposite ones – star *vs.* equal network - led to very different patterns. From a star network to an equal one, the central individual lost its leadership; it had less impact on the joining and then on the decision-making process. This theoretical result was obtained by only taking into account contacts between individuals, and did not include other relationships, such as aggressive and submissive behaviours. This means that a unique leader may emerge without the use of dominance and/or coercion of the dominant individual on subordinate ones. A simple rule based on social network properties allowed us to explain leadership and organizations of group members during movements. We therefore showed a non-linear link between the type of social network and the resulting consensus. The decision-making system switched from an unshared consensus to a shared consensus when the social network switched from a centralized to a decentralized one. By comparing our theoretical data with observed ones, we confirmed that this relationship also seems to exist in groups of primates. The question may appear trivial but this link between social structure and consensus had never been directly demonstrated before this study. Several studies have already attempted to explain the relation between social network and collective decision-making, but tested this link either theoretically or empirically in only one species or *genus* [3], [47], [45]. This study is the first to combine different approaches and to confirm an influence of social network on collective decision-making in a wide range of primate species. These results illustrate that social networks have a great influence on the emergence of leadership and that ecological factors alone cannot explain all the patterns of collective decision-making. This study provides new elements that could help to disentangle contradictory results found on these two hot topics in previous studies [1], [3], [12], [63], [64]. The centrality index is also a new index that can be useful to qualify the level of centralization of a group [32].

The relative importance of affiliative interactions (grooming, contacts) and spatial associations (proximities) is difficult to disentangle, reflecting the fact they are highly correlated in primate societies. In this study, we may wonder whether the emergence of leadership and the type of consensus depend directly on the network or depend more on the spatial distribution of individuals, the decision resulting from a spatial diffusion. However, in the model used in this study, we did not implement the spatial distribution of individuals (they all started from the same point). Moreover, in a study where both spatial and grooming interactions influence the organization and order of group members at departures of collective movements, authors suggested that the influence of grooming was not an artifact of its relationship to spatial association but that individuals do follow ‘friends’ (according to the affiliative relationship), but preferentially those friends that are in closest proximity (according to the spatial distribution depending on the affiliative relationships) [45].

The influence of social networks on collective phenomena has already been described for information or disease transmission [65], [66]. Such authors such as Watts [67], [68] or Fowler [69] already studied the effect of connectivity of networks on the ‘cascade’ propagation on behaviours such as innovations or decisions. Voelkl and Noë [54] used several artificial networks implemented in a multi-agent system to test the influence of social structure on the propagation of social information. They then compared their results with data based on one primate group. In these studies on information transmission, the central individual was then a key element for a higher or lower transmission. Likewise, Sueur and Petit [10], [17] highlighted the fact that the social styles of macaque species influenced how they decided collectively but without directly testing the effect of social network for the different groups. In rhesus macaques, known as a despotic species [31], dominant individuals who are more central increased the probability of other group members joining a movement, whereas in the tolerant Tonkean macaques, each group member had the same weight in the decision-making process [3], [10], [17]. Our theoretical study showed similar results but also that contacts or grooming between individuals can suffice to explain leadership distribution, without any need to take the dominance rank of individuals, their level of aggression or other individual characteristics into account. In a centralized network, the central individual had more joiners and shorter joining latencies than non central individuals did. This result seem to confirm some studies reporting that the dominance individuals, who are often the central ones, were leaders (*Canis lupus* [37]; *Helogale parvula* [38]; *Equus caballu* [39]; *Gorilla gorilla berengei* [40]). On the contrary, in a decentralized, but also in centralized networks with links between non central individuals, there was no difference in patterns (speed of joining and number of joiners) whatever the centrality of the initiator. In this study, we tested the effect of centrality, especially the eigenvector coefficient, on the consensus type. This eigenvector coefficient was also the most common coefficient used to test how information or disease spreads in a group. However, Kitsak et al. [70] theoretically showed that the most efficient individuals allowing the diffusion of information were not always those who were the most connected or had the highest eigenvector centrality coefficient. They found that the most efficient ‘spreaders’ were those located within the core of the network, that is to say the individuals with the highest betweenness coefficient [52], [71]. It should therefore be interesting to study this kind of networks to assess how sub-grouping patterns and betweenness centrality could affect collective decision-making as we did here with the eigenvector centrality.

Recent studies have suggested the existence of unshared consensus in animal groups [72], [73]. This consensus would allow the dominant individual to have better access to food or to satisfy its own needs [11]. In other species, individuals who have the best information for foraging or another activity would also lead the group, whatever their social status. We also showed in this study that an unshared consensus, through the social position of individuals in the network, led to a quicker decision. Nevertheless, Conradt and Roper [1] stipulated that an unshared consensus could lead to high costs for group members because they cannot satisfy their own needs, unlike the leader. This dissatisfaction may drive the group to split, removing all interest for the leader to continue leading the group. We might oppose this to shared consensus, through which all individuals decide to move and can then meet their individual needs. The shared distribution of initiations between all group members also leads to a decrease in the probability of making a mistake regarding the chosen location. Indeed, in the case of a shared decision, information is shared and pooled, which is not the case in unshared consensus [16], [62]. Thus, both kinds of consensus seem to have advantages: speed for an unshared decision versus accuracy for a shared decision. This speed-accuracy trade-off paradigm is well known [74], but to the best of our knowledge, no study has yet attempted to assess whether the social network of a group can affect this trade-off, and therefore also affect the efficiency of a collective decision [2], [29].

Interestingly, we showed that the link between social network and consensus was, however, not linear. Indeed, the relations between the centrality index and the dependent variables follow non-linear functions. The functions we found showed that there was a threshold (about 0.8) where the decision-making system switched from an unshared to a shared consensus. As soon as non central individuals interacted with each other, the consensus turned into a shared consensus. Indeed, the affiliative relationships between all group members seem to play an important role in the decision-making process. The decision-making system appears to be highly non-linear, evolving more rapidly into a mimetic, and especially allelomimetic process (decentralized) than a leadership (i.e. centralized) process [41], [43]. For a long time, scientists believed that group members decided to join a movement due to the leader's departure. However, current results show that animals decide to join a movement according to the number and the identities of all already moving agents, and not only according to the initiator [75].

The present study showed that individuals only took their affiliative relationships into account for moving, and gave particular importance to the strongest relationships. Our model suggested that group members might solely consider local interactions. The central individual had an important role in the decision-making process, but based on simple rules dependant on the network, and especially in this study, on affiliative relationships. Other social interactions, positive or negative, requiring more or less cognitive abilities, did not need to be implemented in our model to understand the emergence of leadership. Authors have suggested that the complexity of collective movement observed in primates – with specific order and associations of individuals – could only emerge thanks to developed cognitive abilities which enable primates to use intentions, manipulations, and insights [12], [14], [76]. Complex phenomena can, however, emerge from simple and local interactions [75]. Hemelrijk [77], [78] showed that the complex spatial positions and associations of macaques – with dominant individuals at the middle of the group and subordinate ones at the periphery – could emerge from simple rules based on how individuals behave after conflicts (staying in the same place or moving away from the winner). Hemelrijk's model, like ours, did not take into account the intentions or cognitive abilities of primates, but successfully reproduced the patterns of the collective phenomena in question.

Our study is a starting point for the investigation of how social networks and consensus are interrelated. Theoretical results were confirmed by observed data in several groups of primates. The five study groups living in semi free-ranging conditions highlighted the importance of the social network rather than the equally important question of ecological pressures. It would be interesting as a second step to recreate the natural environment of animals in order to assess how ecology and social network interact together to constrain consensus decisions. How social network influences decisions is a crucial question and answering this question may have direct applications as management and conservation of animal populations [79], [80] or in firm management and Economics [81].

## Supporting Information

Figure S1 **Graph representation of social networks.** (a) Erdos-Renyi random network and (b) chain network. Squares represent individuals. *C* is the central individual, *c_1–9_* are non central individuals. Lines are relationships between individuals: the thicker the line, the stronger the relationship. The size of square represents the eigenvector centrality: the bigger the square, the higher the centrality.
(TIF)

Figure S2 **Mean departure latency according to the rank for each social network.**
(TIFF)

Figure S3 **Latencies' distribution of joiners when the initiator is the central individual (blue) and when it is a non central individual (red) for each social network.**
(TIFF)

Table S1 **Relationships, eigenvector centrality for the central individual** ***C*** **and the non central individuals** ***c*** **, and centrality index for the random network and the chain network.**
(DOC)

Table S2 **Detailed results for Dunn's multiple comparison test.** *** : p<0.0001; **: p<0.01; *: p<0.05.
(DOC)
