# Supplementary figures and images for: From Social Network (Centralized vs. Decentralized) to Collective Decision-Making (Unshared vs. Shared Consensus)
Source: PLoS One. 2012 Feb 29;7(2):e32566. doi: 10.1371/journal.pone.0032566 (PMC3290558; doi:10.1371/journal.pone.0032566)

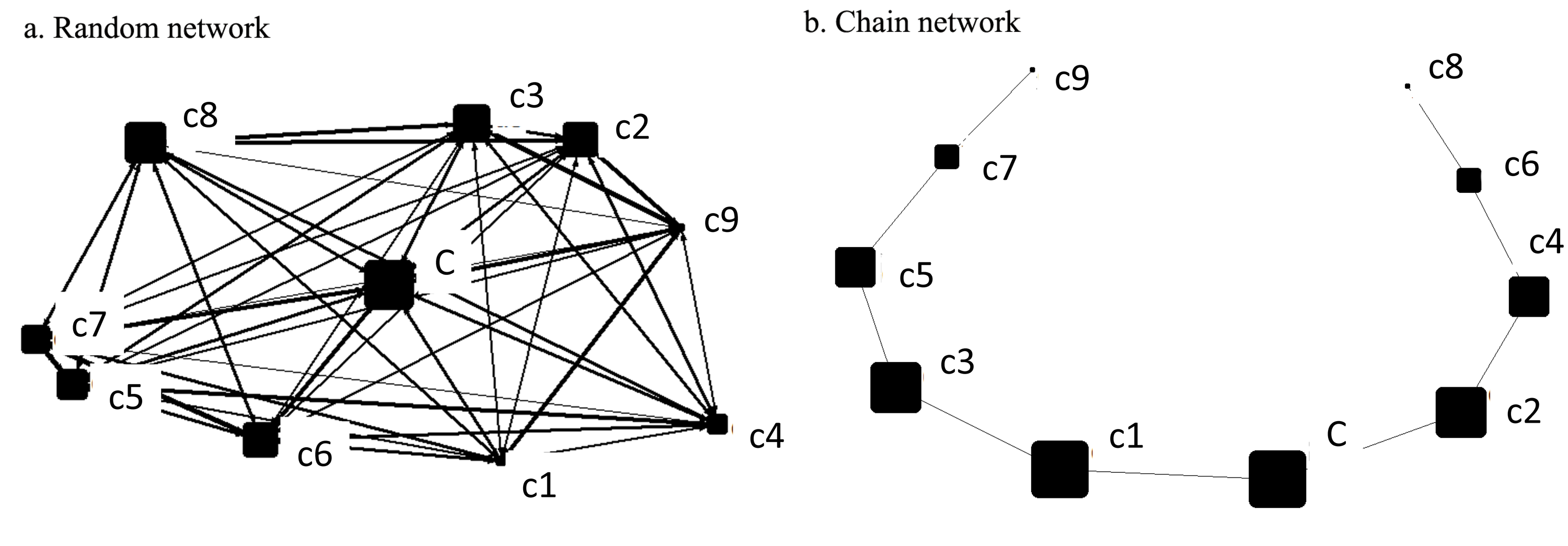

Supplement: Figure S1 — Graph representation of social networks. (a) Erdos-Renyi random network and (b) chain network. Squares represent individuals. C is the central individual, c1–9 are non central individuals. Lines are relationships between individuals: the thicker the line, the stronger the relationship. The size of square represents the eigenvector centrality: the bigger the square, the higher the centrality. (TIF) [file pone.0032566.s001.tif]

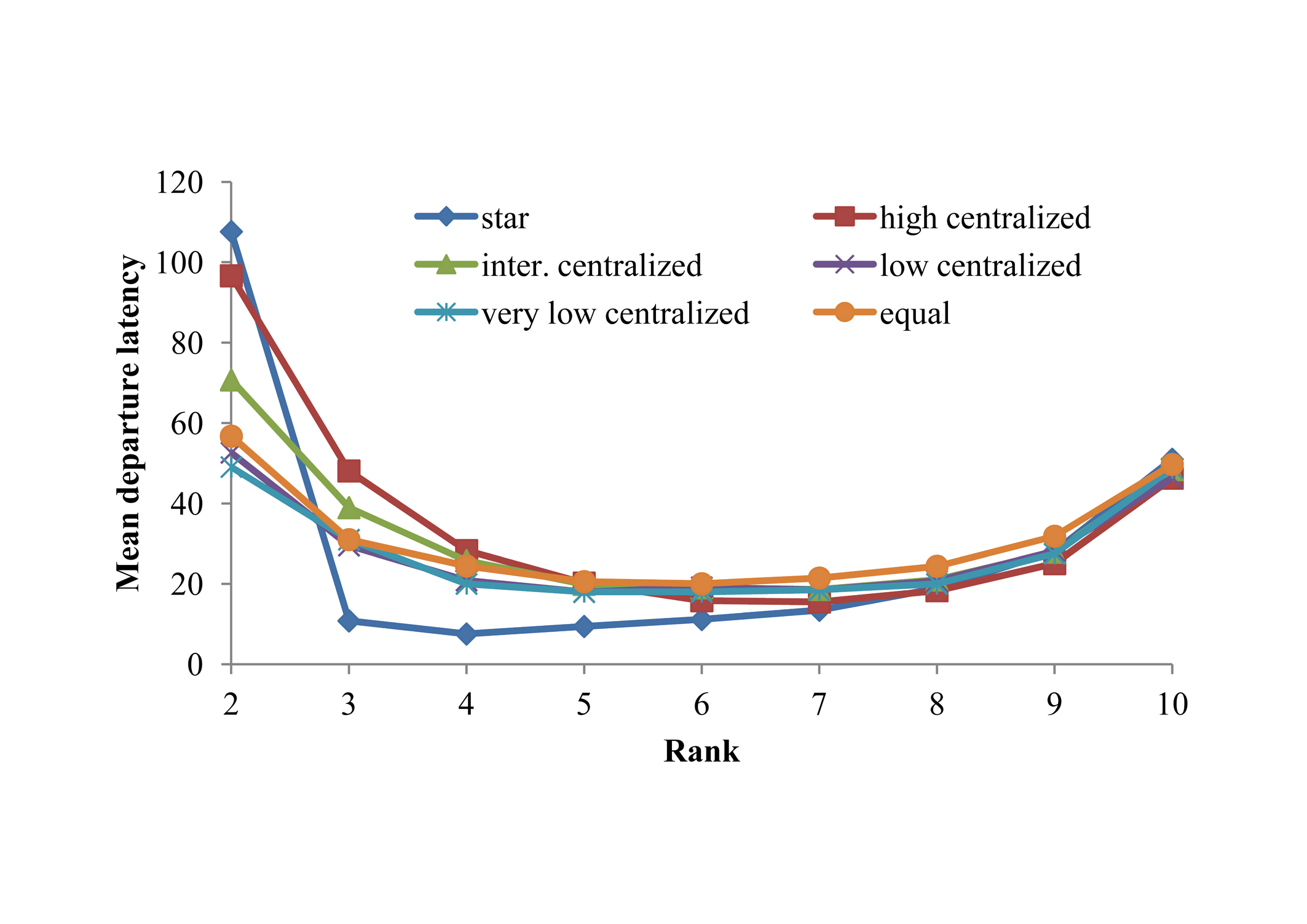

Supplement: Figure S2 — Mean departure latency according to the rank for each social network. (TIFF) [file pone.0032566.s002.tiff]

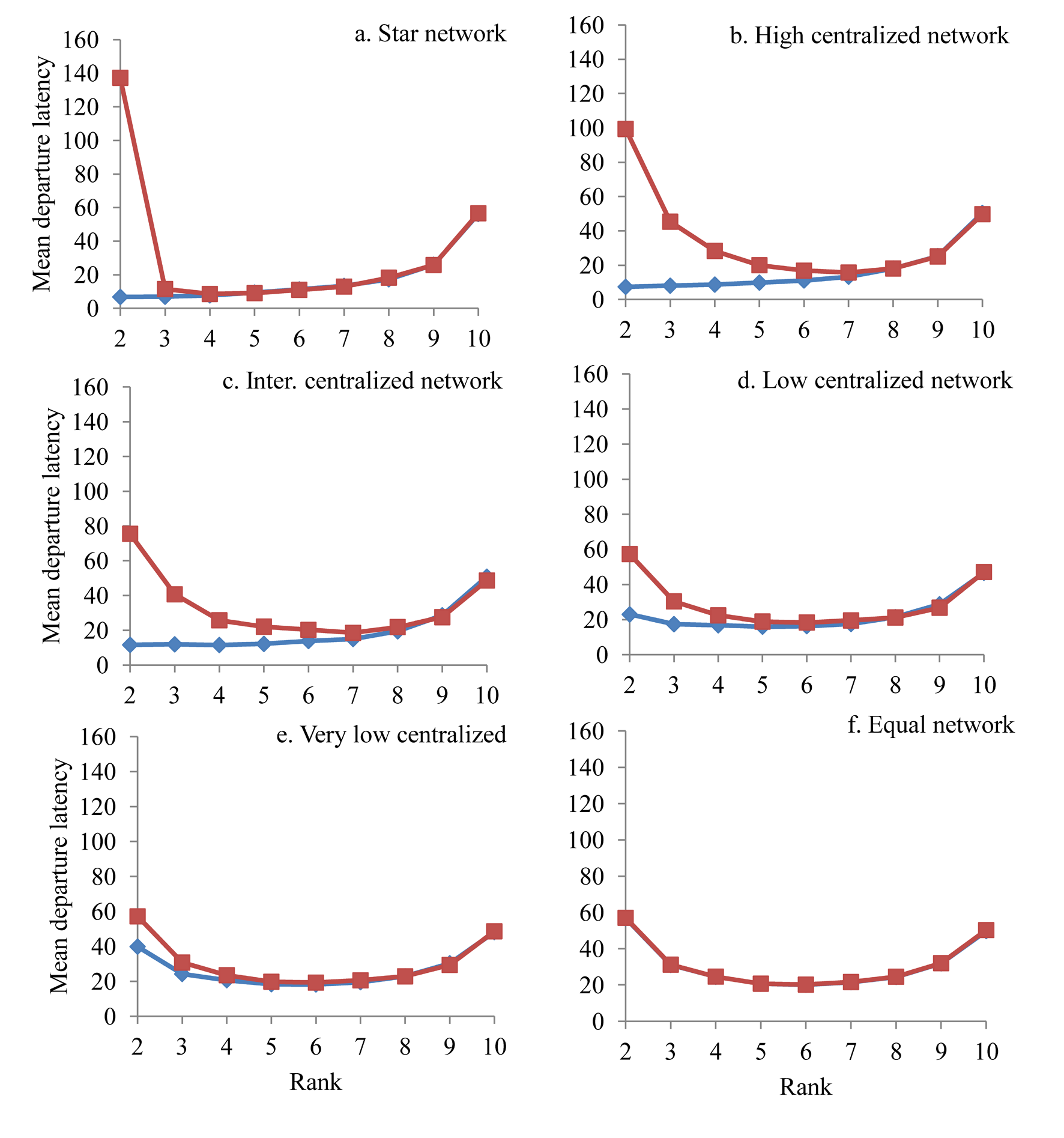

Supplement: Figure S3 — Latencies' distribution of joiners when the initiator is the central individual (blue) and when it is a non central individual (red) for each social network. (TIFF) [file pone.0032566.s003.tiff]
