# Supplementary material for: From Social Network (Centralized vs. Decentralized) to Collective Decision-Making (Unshared vs. Shared Consensus)
Source: PLoS One. 2012 Feb 29;7(2):e32566. doi: 10.1371/journal.pone.0032566 (PMC3290558; doi:10.1371/journal.pone.0032566)
Supplement: Table S1 — Relationships, eigenvector centrality for the central individual C and the non central individuals c , and centrality index for the random network and the chain network. (DOC) [file pone.0032566.s004.doc]

Table S1: Relationships, eigenvector centrality for the central individual *C* and the non central individuals *c*, and centrality index for the random network and the chain network.

| Network | r(c, C) | r(c, c) | r(C, c) | Eigenvector of C | Eigenvector of c | Centrality index |
| --- | --- | --- | --- | --- | --- | --- |
| Random | ]0,1[; > r(c, c) | ]0,1[; < r(c, C) | ]0,1[ | 0.39 | 0.344±0.015 | 0.085 |
| Chain | - | - | - | 0.42 | 0.283±0.038 | 0.12 |
