# Supplementary material for: From Social Network (Centralized vs. Decentralized) to Collective Decision-Making (Unshared vs. Shared Consensus)
Source: PLoS One. 2012 Feb 29;7(2):e32566. doi: 10.1371/journal.pone.0032566 (PMC3290558; doi:10.1371/journal.pone.0032566)
Supplement: Table S2 — Detailed results for Dunn's multiple comparison test. *** : p<0.0001; **: p<0.01; *: p<0.05. (DOC) [file pone.0032566.s005.doc]

Table S2: detailed results for Dunn’s multiple comparison test. *** : p < 0.0001; **: p < 0.01; *: p < 0.05.

| Dunn's Multiple Comparison Test | Mean number of joiners | Mean latency of the 1st joiner | Mean duration of joining |
| --- | --- | --- | --- |
| very low centr. network vs. equal network | ns | ** | *** |
| low centralized network vs. equal network | ns | ns | *** |
| low centralized network vs. very low cent. network | ns | ns | ns |
| inter. centralized network vs. equal network | ns | ** | ns |
| inter. centr. network vs. very low cent. net. | ns | *** | *** |
| interm. centr. network vs. low centr. network | ns | *** | *** |
| highly centralized network vs. equal network | *** | *** | * |
| highly centr. network vs. very low cent. network | *** | *** | *** |
| highly centr. network vs. low centralized network | *** | *** | *** |
| highly centr. network vs. inter. centr. network | *** | *** | ns |
| star network vs. equal network | *** | *** | *** |
| star network vs. very low centralized network | *** | *** | ns |
| star network vs. low centralized network | *** | *** | ns |
| star network vs. intermediate centralized network | *** | *** | *** |
| star network vs. highly centralized network | *** | ns | *** |
| random network vs. equal network | ns | ** | *** |
| chain network vs. equal network | *** | ns | *** |
| random network vs. very low cent. network | ns | ns | ns |
| chain network vs. very low cent. network | *** | ns | *** |
| random network vs. low centralized network | ns | ns | ns |
| chain network vs. low centralized network | *** | ns | *** |
| random network vs. inter. centralized network | ns | *** | *** |
| chain network vs. inter. centralized network | *** | *** | *** |
| random network vs. highly centralized network | *** | *** | *** |
| chain network vs. highly centralized network | *** | *** | *** |
| random network vs. star network | *** | *** | ns |
| chain network vs. star network | *** | *** | *** |
